# Antioxidant Phytochemicals in Fresh Produce: Exploitation of Genotype Variation and Advancements in Analytical Protocols

**DOI:** 10.3389/fchem.2017.00095

**Published:** 2018-02-06

**Authors:** George A. Manganaris, Vlasios Goulas, Ifigeneia Mellidou, Pavlina Drogoudi

**Affiliations:** ^1^Department of Agricultural Sciences, Biotechnology and Food Science, Cyprus University of Technology, Lemesos, Cyprus; ^2^Hellenic Agricultural Organization ‘Demeter’, Institute of Plant Breeding and Genetic Resources, Thessaloniki, Greece; ^3^Hellenic Agricultural Organization ‘Demeter’, Department of Deciduous Fruit Trees, Institute of Plant Breeding and Genetic Resources, Naoussa, Greece

**Keywords:** ascorbic acid, carotenoids, polyphenols, phytochemicals, reactive oxygen species, spectroscopic methods, landrace, traditional cultivars

## Abstract

Horticultural commodities (fruit and vegetables) are the major dietary source of several bioactive compounds of high nutraceutical value for humans, including polyphenols, carotenoids and vitamins. The aim of the current review was dual. Firstly, toward the eventual enhancement of horticultural crops with bio-functional compounds, the natural genetic variation in antioxidants found in different species and cultivars/genotypes is underlined. Notably, some landraces and/or traditional cultivars have been characterized by substantially higher phytochemical content, i.e., small tomato of Santorini island (cv. “Tomataki Santorinis”) possesses appreciably high amounts of ascorbic acid (AsA). The systematic screening of key bioactive compounds in a wide range of germplasm for the identification of promising genotypes and the restoration of key gene fractions from wild species and landraces may help in reducing the loss of agro-biodiversity, creating a healthier “gene pool” as the basis of future adaptation. Toward this direction, large scale comparative studies in different cultivars/genotypes of a given species provide useful insights about the ones of higher nutritional value. Secondly, the advancements in the employment of analytical techniques to determine the antioxidant potential through a convenient, easy and fast way are outlined. Such analytical techniques include electron paramagnetic resonance (EPR) and infrared (IR) spectroscopy, electrochemical, and chemometric methods, flow injection analysis (FIA), optical sensors, and high resolution screening (HRS). Taking into consideration that fruits and vegetables are complex mixtures of water- and lipid-soluble antioxidants, the exploitation of chemometrics to develop “omics” platforms (i.e., metabolomics, foodomics) is a promising tool for researchers to decode and/or predict antioxidant activity of fresh produce. For industry, the use of optical sensors and IR spectroscopy is recommended to estimate the antioxidant activity rapidly and at low cost, although legislation does not allow its correlation with health claims.

## Introduction

The increased dietary intake of fresh produce has been a fundamental element of public health policy for the past years, as has been long considered to provide protection against a wide range of oxidative-stress related diseases, such as cancer, stroke, diabetes, Alzheimer's, cataract, and age-related function decline (Arts and Hollman, 2005). All recent studies support that a diet rich in fruit and vegetables is beneficial for human health, suggesting that these key bioactive compounds should be a clear target for enhancement of nutritional value of fresh produce.

Low amounts of reactive oxygen species (ROS) are continuously generated in cells of aerobic organisms (Kitsati et al., 2012), and play, as unequivocally accepted, important physiological roles. However, when ROS levels increase, an unregulated oxidation of cell components may be induced, a fact that is directly linked with the initiation and/or progression of an array of pathological conditions. Such conditions can be combated or delayed through the consumption of exogenous protective compounds (Kitsati et al., 2012). Epidemiological studies also support that the consumption of fresh produce, containing phenolic and non-phenolic antioxidants is inversely associated with risk of chronic diseases (Naska and Trichopoulou, 2014). In this context, the antioxidant potency *per se* is a well-established biomarker, which indicates beneficial biological effects of fresh produce.

In the sections below, the significance of exploiting the natural genetic variation, including landraces, traditional and commercial cultivars, for the enhancement of horticultural crops with bio-functional compounds is discussed. Considering the vast amount of genotypes for a given horticultural commodity and their different behavior under diverse ecological environments, there is an urgent need to screen and precisely evaluate their antioxidant capacity and overall phytochemical content in a compatible manner with up-to-date analytical approaches. Toward this aim and in view of the fact that a great number of methods for valorizing antioxidant capacity has been proposed over the recent years, this review focuses in techniques that can fulfill the requirements of horticulturists and food scientists for rapid, easy and low cost analytical tools.

## Exploitation of genotype variation to enhance bio-functional compounds

A wide diversity in the concentrations of key bioactive compounds (especially ascorbic acid (AsA), phenypropanoids and carotenoids) among cultivars within the same species has been recorded (reviewed in Kanellis and Manganaris, 2014). It is therefore conceivable that screening studies on genotypic diversity are of prime importance, especially in horticultural crops where the production is derived from a significant number of cultivars, i.e., peach, sweet cherry, apple. Although factors, such as environmental conditions (Dumas et al., 2003), developmental/ripening stage (Hancock et al., 2007; Ioannidi et al., 2009; Drogoudi and Pantelidis, 2011; Ilahy et al., 2011; Liu et al., 2015), tissue type (Drogoudi et al., 2007; Bulley et al., 2009), and pre- and post-harvest treatments (Ioannidi et al., 2009) play a pivotal role in regulating the accumulation of bioactive molecules in fresh commodities, genetic control appears to be by far the dominant source of variation within a species. As traits linked to nutritional quality are usually under polygenic control and quantitative inheritance, understanding the existing natural diversity, including indigenous and/or traditional cultivars at the genetic level is a great challenge in modern breeding programs. Early domestication and modern breeding strategies has led to the loss of certain genome regions linked to stress resistance and/or nutritional value, usually in favor of crop yield (Fernie et al., 2006). Ascorbic acid, polyphenols and carotenoids constitutes main antioxidant classes and are analyzed in detail in the following sections.

### Ascorbic acid

Horticultural commodities, such as pepper, strawberry, kiwifruit and citrus possess significant amounts of AsA, whereas other popular in human diet species, such as tomato and apple contain moderate amounts. The accumulation of AsA may vary not only within different species, but also among different cultivars (Bulley et al., 2009; Mellidou et al., 2012a,b; Gest et al., 2013), tissue types, with photosynthetically-active tissues containing higher levels than heterotrophic ones (Bulley et al., 2009) and developmental stage (Hancock et al., 2007; Bulley et al., 2009; Ioannidi et al., 2009; Mellidou et al., 2012b) within the same species. Indicatively, AsA content of edible parts of commercial cultivars may substantially vary both within commercial cultivars and germplasm (Table 1). Intra-variety diversity in AsA content due to population × year interaction or due to the different sampling locations may also be critical in several fruit species, such as tomato (Cortés-Olmos et al., 2014) and apple (Pissard et al., 2012), pointing out the urgent need to develop robust protocols to analyze fruit quality.

**Table 1:** Variability in ascorbic acid (AsA) content within commercial cultivars and germplasm.

**Fold-Range in AsA Content**			
**Commodity**	**Commercial cultivars**	**Germplasm**	**References**
Kiwifruit	2	20	Davey et al., 2000; Gest et al., 2013
Strawberry	1.6	2.3	Davey et al., 2000; Cruz-Rus et al., 2011
Tomato	2.5	9.0	Mellidou et al., 2012a; Gest et al., 2013
Peach	7.7	–	Davey et al., 2000
Apple	6.5	13	Mellidou et al., 2012b; Gest et al., 2013
Grape	1.9	3.3	Melino et al., 2009; Gest et al., 2013

An excellent example to explore genotype variation and to elucidate how the differences in AsA metabolic pathways and the genes involved therein govern the AsA pool is the landrace “Santorini” a drought-tolerant tomato cultivar with high fruit AsA content, indigenous to Santorini island and protected designation of origin (PDO) product in Greece (Mellidou et al., 2012b; Koutsika-Sotiriou et al., 2016). Integration of metabolite analyses, non-labeled, and radio-labeled substrate feeding experiments, enzyme activity and transcript level profiling showed that enhanced AsA recycling activities were responsible for AsA accumulation during the last stages of ripening (Mellidou et al., 2012b).

Apart from differences between commercial cultivars, an interesting observation is that AsA concentrations tend to be higher in wild accessions compared to modern cultivars (Table 1). For example, within tomato germplasm, *Solanum pennellii*, and *S. lycopersicum* cv cerasiforme contain 5- and 2-fold more AsA, respectively, than the traditional cultivars of *S. lycopersicum* (Stevens et al., 2007), while natural diversity may reach up to 9-fold (Gest et al., 2013). Presumably, this is due to the fact that during plant evolution, traits, such as fruit size and total plant yield have been selected at the cost of other parameters, such as vitamin C, as modern crops are usually cultivated in favorable environments at the absence of stress conditions. Indeed, comparative studies between *S. lycopersicum* and *S. pennellii*, revealed that the wild accession responds much more efficiently to low-water conditions (Stevens et al., 2008). Cherry tomatoes are considered as intermediate genotypes both genetically and phenotypically between wild and cultivated tomatoes, and are richer in AsA than their large fruited counterparts. Additionally to the dilution effect that leads the decrease of AsA pool size, there seems to be a clear genetic linkage between fruit size and AsA concentrations, as revealed by the collocation of major Quantitative Trait Locus (QTL) for AsA contents with QTLs for fruit weight in both apple (Mellidou et al., 2012a) and tomato (Stevens et al., 2007). Whether domestication, that systematically favored growth at the expense of accumulating other necessary metabolites, has also influenced AsA metabolic pathways at the protein (structure) or gene (expression patterns) or gene cellular compartmentation level (Gest et al., 2013), remains an open question that merits further investigation.

Such differences within a species may be primarily attributed to higher rates of net biosynthesis, recycling, and/or intercellular and intracellular transport, or decreased turnover capacity, and feedback regulation of AsA pool through inhibition of the activity of the last enzyme in the biosynthetic pathway. Although numerous studies on genetic factors demonstrated that AsA accumulation showed a relatively high heritability, it also seems to be highly responsive to environmental stimuli and post-harvest treatments (Mellidou and Kanellis, 2017). The pathway derived from _L_-galactose is generally considered as the dominant route of AsA biosynthesis in *Arabidopsis* (Bulley et al., 2009; Yoshimura et al., 2014), tomato (Gilbert et al., 2009; Ioannidi et al., 2009; Bulley et al., 2012; Mellidou et al., 2012b; Wang et al., 2013), kiwifruit (Bulley et al., 2009; Li et al., 2010), black currant (Hancock et al., 2007), citrus (Alós et al., 2014), blueberry (Liu et al., 2015), and potato tuber (Bulley et al., 2012).

Several lines of evidence suggest GDP-_L_-galactose phosphorylase (GGP), the enzyme catalyzing the first committed step of the pathway is the key control point in the AsA biosynthetic pathway, and its transcription has been correlated with the AsA pool size in several species (Bulley and Laing, 2016; Mellidou and Kanellis, 2017). Although the transcriptional control of *GGP* in regulating AsA pool size is well-established, it is only recently that its translational regulation under unfavorable conditions has been also elucidated (Laing et al., 2015). The proposed model is based on the identification of a highly conserved non-canonical upstream open reading frame (uORF) in a wide range of species that allows the feedback regulation of GGP translation under rapidly changing conditions, without the need of gene transcription modifications. Allelic associations studies in a mapping population and 22 commercial cultivars of apple reinforce the notion that SNPs found in *GGP* coding sequence are rather linked to polymorphisms in the promoter region that alter allele expression, than to altered protein function (Mellidou et al., 2012a).

The genetic regulation of AsA accumulation may also vary depending on the tissue or developmental stage considered. Toward this end, in red ripe tomato fruit, the alternative routes proceeding either via _D_-galacturonate (Badejo et al., 2012) or via *myo*-inositol (Mellidou et al., 2012b) may also contribute to an enhanced AsA pool size in certain genotypes, presumably as a tool to support biosynthesis via the main route under unfavorable conditions. Furthermore, the alternative biosynthetic pathway via uronic acids has been suggested to exert a major role in regulating fruit AsA content in strawberry (Agius et al., 2003; Cruz-Rus et al., 2011), citrus (Xu et al., 2012), and rose (Li et al., 2017). Apart from changes in AsA biosynthetic capacity, perturbations in the AsA recycling pathway by altering AsA redox homeostasis may also alter not only AsA concentrations, but also plant responses to various environmental stimuli (Foyer and Noctor, 2011; Fotopoulos and Kanellis, 2013). Although a plethora of classical and modern breeding strategies have been employed to unravel the genetic mechanisms underlying AsA accumulation, a lot of effort is still required to restore its levels to cultivated crops toward producing high-quality and sustainable horticultural commodities for future generations.

### Polyphenols

Polyphenols are the most abundant bioactive compounds with antioxidant activity, and consist of a wide range of biomolecules, such as simple phenols, phenolic acids, flavonoids, polymeric phenolics, and anthocyanins (Kanellis and Manganaris, 2014). Based on the structure of their basic skeleton, the main phenolic compounds are phenolic acids, flavonoids and anthocyanins (Lima et al., 2014). Phenol-explore database summarizes more than 35,000 content values for 500 different polyphenols in over 400 foods (http://phenol-explorer.eu/). Anthocyanins are vacuolar pigments usually present in the skin of fruits responsible for fruit color in several species (e.g., berries, grape, cherry, pomegranate, plum, apple) giving their characteristic red, blue or purple pigmentation. Other compounds of this category that are less studied in horticultural crops are lignans, stilbenes, tannins, coumarins, and lignins. In line with findings on AsA, landraces and unexplored cultivars accumulate such compounds at high levels providing a valuable tool toward sustainable breeding for the development of nutritionally-enriched cultivars. On the other hand, high concentrations of TPs may result to unpalatable bitter or astringent tastes (Lesschaeve and Noble, 2005) that may or not be related to browning coloration in fruit and vegetables (Cantín et al., 2011; Saeed et al., 2014). In particular, soft fruits (strawberry, blackberry, blueberry, raspberry) are generally characterized by appreciably high antioxidant potential but such fruit cannot be widely available due to short shelf-life or consumption is limited due to undesirable organoleptic characteristics being bitter, sour or astringent (i.e., aronia, cornelian cherry, or hyppophaes) (reviewed in Manganaris et al., 2014).

Apple (*Malus* × *domestica*) is the most important temperate fruit crop. Notably, high genetic variability in polyphenolic compounds within apple germplasm exist. Comparing wild germplasm with commercial cultivars, total polyphenolic content was found to vary significantly within *Malus sieversii* and *Malus* × *domestica* germplasm (up to 9-fold in the flesh, and up to 4- in the peel), with the wild genotypes of *Malus sieversii* generally containing higher concentrations in individual polyphenol groups (Volz and McGhie, 2011). Breeding and germplasm conservation strategies aim to improve polyphenolic for enhancing quality traits related to phenylpropanoid pathway (color, aroma, disease resistance, and browning disorders) of elite modern cultivars (Wojdyło et al., 2008).

The availability of the draft apple genome sequence (Velasco et al., 2010) caused a boost in apple genetics providing new tools for candidate gene detection. Over 50 metabolites, peel- and flesh-related, from the phenylpropanoid pathway were found to collocate with a certain QTL hotspot in linkage group 16, that contains a structural gene of the pathway, leucoanthocyanidin reductase (*LAR*), indicating that this gene may play a pivotal role in regulating the pathway (Khan et al., 2012). A feedback regulation of proanthocyanidin (PA) biosynthesis has been proposed, according to which low PA levels may enhance the transcription of structural biosynthetic genes of the pathways, such as LAR (Henry-Kirk et al., 2012).

Notably, over the last decade, red pigmentation of apples has received considerable attention and new red-fleshed apple cultivars have been released into the market, being considered of higher nutritional value (high anthocyanin levels). Cyanidin 3-*O*-galactoside is the dominant anthocyanin in both flesh and skin (Volz et al., 2014). Based on recent advances, anthocyanin accumulation can be regulated by three main mechanisms: (1) the anthocyanin biosynthetic pathway, (2) the transcription regulation through the MYB protein that affects anthocyanin biosynthesis during fruit growth and development, and (3) the anthocyanin transport from cytosol where it is synthesized to the vacuole where it is stored (Espley et al., 2009; Hu et al., 2016). Stable QTLs for polyphenolic content have been identified in apples and SNP-derived markers have been developed for polyphenolic-rich cultivars (Chagné et al., 2012, 2016). Using a transgenic/cisgenic approach, the integration of the dominant red-flesh MYB allele of the transcription factor MYB10 resulted in apples with significantly higher levels of foliar, flower and fruit (especially in the peel) anthocyanins in the transgenic red-fleshed apples (Espley et al., 2013).

Except for apple, a significant diversity in phenolic compounds among different genotypes of commodities of high nutritional value, such as soft fruits (Stephens et al., 2009; Stevenson and Scalzo, 2012) and pomegranates has been recorded. “Wonderful” and “Hicaznar” global pomegranate cultivars possessed higher total phenolic concentrations compared to local cultivars, grown in Spain (Legua et al., 2016). This is the case also for other commercially important fruit commodities, as apricot (Sochor et al., 2011), and peach (Kwon et al., 2015). Notably, the reference nectarine cultivar “Big Top,” that is highly appreciated by the consumers due to exceptional qualitative attributes, was characterized by low antioxidant capacity (Drogoudi et al., 2016). Late-harvested compared to early- harvested cultivars, tended to be characterized by higher polyphenolic contents (Font i Forcada et al., 2014; Drogoudi et al., 2016).

### Carotenoids

Carotenoids are lipophilic molecules responsible for the yellow, orange and red pigmentation of several fruit. There are two main groups of carotenoids, one being carotenes [α-carotene, β-carotene (the precursor of vitamin A) and lycopene the most powerful antioxidant among this group], the other being oxygenated derivatives known as xanthophylls (lutein, cryptoxanthin, violaxnthin, and zeaxanthin). Generally, carrots, sweet potato, pineapple, mango, peach, and tomato are rich in carotenoids. The latter, taking additionally into account its increased consumption, is considered as a valuable source of lycopene and β-carotene in the human diet (Fraser and Bramley, 2004). Furthermore, tomato is widely used as a model species for carotenoid studies due to its diverse fruit pigmentation (yellow, orange, orange-red, and red) with different carotenoid profiles (Yuan et al., 2015). For instance, red tomatoes are rich in lycopene, while orange fruits of *Beta* mutants contain high β-carotene levels but at the expense of lycopene. Similar to findings on AsA, carotenoids are highly accumulated in wild species, such as *S. pennellii* compared to their domesticated relatives (Fernie et al., 2006). A dominant allele from wild species has been found to remarkably increase β-carotene in the fruit via higher β-cyclase activity (Ronen et al., 2000). Several studies on tomato natural mutants and transgenic plants with altered carotenoid accumulation allowed to decipher the complete carotenoid biosynthetic pathway in tomato (reviewed in Martí et al., 2016).

As far as other horticultural crops are concerned, *Citrus* spp. have the greater variation in carotenoid contents among different species and cultivars. For example orange, mandarin and clementine fruit contain high amounts of β-cryptoxanthin, violaxanthin, lutein and zeaxanthin, while lemon, and lime are relatively poor in carotenoids (Yuan et al., 2015). A wide diversity (up to 25-fold) between commercial cultivars and/or accessions has also been reported in *Musa* spp. (Davey et al., 2009; Borges et al., 2014). The commercially important bananas of the Cavendish group do not contain high levels of carotenoids, whilst other genotypes identified in banana germplasm contain significant amounts of these compounds (Amorim et al., 2009; Davey et al., 2009). Although factors, such as the degree of maturation, the environment, the type of soil, and storage conditions may be important in regulating carotenoids contents in banana, the most significant factors appears to be the genotype and the genetic origin (Davey et al., 2007; Amorim et al., 2009). Generally, genotypes from the genomic group AAB are rich in carotenoid levels compared to those from the AAA group (i.e., Cavendish), probably due to the presence of the B genome (Davey et al., 2007), indicating the potential of carotenoid compounds to be successfully bio-fortified in breeding programs.

Multidisciplinary research programs currently focus on bio-fortification of carotenoids in fruits and vegetables, mainly targeting on β-carotene, in an effort to tackle vitamin A deficiency (Farré et al., 2011). Therefore, understanding the genetic mechanisms underlying carotenoid accumulation responsible for the genetic diversity across and within species is a great challenge. Carotenoids and especially those having pro-vitamin A activity are quantitative traits, and their contents are regulated by the activity of multiple gene products (Davey et al., 2009). Carotenoid biosynthesis is now well-established with most of the genes being characterized in many species (Lu and Li, 2008; Cazzonelli and Pogson, 2010; Martí et al., 2016). Overall, precursor substrate availability (isopentenyl diphosphate and dimethylallyl-diphosphate), as well as the accumulation of phytoene via phytoene synthase (*PSY*) and the phytoene desaturase, are considered as key limiting factors in regulating carotenoid contents in various species (Rodríguez-Villalón et al., 2009; Ravel et al., 2013; Jourdan et al., 2015). Increasing the expression of *PSY* and lycopene β-cyclase (*LYCB*) in tomato has led to significant enhancement of the level of β-carotene and lycopene, respectively (Fraser et al., 2002; D'Ambrosio et al., 2004). Interestingly, when *LYCB* is overexpressed, the levels of β-carotene are also increased as seen in Golden Rice (Ye et al., 2000), due to the shift of the metabolic balance from the α to the β branch of carotenoid pathway at the expense of α-carotene and lutein (D'Ambrosio et al., 2004; Farré et al., 2011). In carrot, polymorphism associated with zeaxanthin epoxidase (*ZEP*) was associated with carotenoid and phytoene contents, indicating that this is a key gene governing carotenoid accumulation in carrot roots. Toward this end, the *high-pigment 3* tomato mutants (mutation occurring on *ZEP* gene) had 30% more carotenoids in mature fruit (Galpaz et al., 2008). There is also compelling evidence that several regulatory genes and proteins as well as a large number of transcription factors affect the hormonal control of carotenoid accumulation at diverse levels through regulation of fruit ripening. In this regard, tomato mutants, such as *rin* and *nor* deficient in ethylene synthesis or perception not only show delayed normal ripening but also modified carotenoid content (Martí et al., 2016). Consistently, transcript levels of *PSY* appeared to be regulated by ethylene, whilst the ethylene-response transcription factor ERF6 plays a vital role in carotenoid accumulation in tomato fruit (Lee et al., 2012).

## Advanced analytical techniques to determine antioxidant potential

Bond dissociation energy and ionization potential are two major factors that determine the mechanism and the efficiency of antioxidants (Karadag et al., 2009). Thus, hydrogen atom transfer (HAT) and electron transfer (ET) reactions are the chemical principles of most widely employed antioxidant activity assays. Furthermore, antioxidant activity analyses can be also classified into redox potential and reactive species scavenging assays. In this context, a great number of simple spectrophotometric tests have been developed. The most commonly applied are 1,1-diphenyl-2-picryl-hydrazyl (DPPH) assay, ferric reducing/antioxidant power (FRAP), ABTS radical cation decolorization, oxygen radical absorbance capacity (ORAC), phosphomolybdenum, cupric ion reducing antioxidant capacity (CUPRAC) and superoxide radical scavenging activity. Due to great importance of antioxidants for human health, a panel of methods to determine the antioxidant activity of fresh produce in biological systems has been also applied, such as inhibition of LDL-cholesterol and of DNA oxidation as well as DNA nicking, haemolysis inhibition and cellular antioxidant capacity (Shahidi and Zhong, 2015).

Taking into consideration that fruit and vegetables are complex mixtures of natural antioxidants, many analytical techniques has been developed to assess their antioxidant activity. In particular, spectroscopy, and electrochemistry are widely used for the determination of antioxidant activity of horticultural produce. Further, flow injection analysis (FIA), optical sensors and high resolution screenings (HRS) allow the rapid estimation of antioxidant activity; whereas chemometrics predict the antioxidant activity extracting data from classical analytical chemistry. All these approaches pursue to reduce time of analysis and the use of harmful reagents and to automate the determinations. The main drawback of these methods as for simple colorimetric assays is the need to prepare extracts in order to measure antioxidant activity. Table 2 summarizes the aforementioned analytical methods, providing their application potential for the determination of antioxidant activity in fresh produce.

**Table 2:** A brief overview of analytical methods for the determination of antioxidant activity in fresh produce.

**Mechanism of measurement**	**Instrumentation/ Statistics**	**Applications**	**References**
**INFRARED (IR) SPECTROSCOPY**			
Characteristic bands in the infrared region of spectra are attributed to the hydroxyl and phenolic groups. These bands are mainly used to predict the antioxidant activity with the employment predictive models.	FTIR^a^ spectrometer/Partial least squares regression (PLSR) models	prediction DPPH^b^, FRAP^c^, TEAC^d^ activity in onion, shallot and garlic	Lu et al., 2011a,b
	NIR^e^ spectrometer/principal component regression (PCR) model	Prediction TEAC activity in green tea	Zhang et al., 2004
	FTIR spectrometer/PLSR models	Prediction ORAC^f^ activity in small fruits	Lam et al., 2015
**ELECTRON PARAMAGNETIC RESONANCE (EPR) SPECTROSCOPY**			
EPR spectroscopy monitors the scavenging of stable free radicals (ABTS^g^, DPPH, hydroxyl radicals) by antioxidants.	EPR spectrometer	Measurement TEAC activity in thymes	Orłowska et al., 2015
		Determination DPPH and TEAC activity in corozo fruit	Osorio et al., 2011
		Measurement of DPPH activity in basil lipophilic extracts	Sgherri et al., 2011
**ELECTROCHEMICAL METHODS**			
Cyclic voltammetry and differential pulse voltammetry measure redox potential of antioxidants	Potentiostat/galvanostat, closed standard three electrode cell, glassy carbon electrode	Measurement antioxidant activity in wild plants	Barros et al., 2011
	Three-electrode electrochemical cell equipped with working electrode, platinum wire auxiliary electrode and Ag/AgCl/sat. KCl reference electrode, glassy carbon electrode	Measurement antioxidant activity in small fruits	Cata et al., 2016
**FLOW INJECTION ANALYSIS (FIA) METHODS**			
The FRAP assay adapted to the FIA system	Peristaltic pump, loops, reactor, flow cell, UV spectrometer	Determination of FRAP activity in tea	Martins et al., 2013
The measurement is based on the inhibition effect of samples on the Co(II)/EDTA^h^-induced luminol-perborate chemiluminescence. The assay adapted to the FIA system.	Peristaltic pump, loops, flow cell, injection valve, Chemiluminescence detector	Determination of TEAC activity in tea infusions, wines, and grape seeds	Pulgarín et al., 2012
This method is based on the transient negative signal measurements with a flow-type platinum electrode detector due to the composition change of a [Fe(CN)_6_]_3_-/[Fe(CN)_6_]_4_- redox-reagent solution.	Peristaltic pump, loops, reactor, flow cell, platinum electrode detector	Determination of antioxidant activity in plant extracts	Shpigun et al., 2006
**OPTICAL SENSORS**			
Optical sensor membranes based on immobilized chromogenic radicals (DPPH) for the assessment of antioxidant activity	UV spectrometer	Determination DPPH activity in various beverages and foods	Steinberg and Milardović, 2007
The chromogenic redox reagent (copper(II)-neocuproine complex) was immobilized onto a cationexchanger film of Nafion.	UV spectrometer	Determination CUPRAC^i^ activity in food extracts	Bener et al., 2010
The measurement capitalizes on the on-paper nucleation of gold ions to its respective nanoparticles, upon reduction by antioxidant compounds present in sample.	Scanner or camera or smartphone	Measuring antioxidant potential in foods	Choleva et al., 2015
**HIGH RESOLUTION SCREENING METHODS**			
The determination of antioxidants was based on a decrease in absorbance after post-column reaction of HPLC-separated antioxidants with the radicals (DPPH or ABTS). Each of the antioxidants separated by the HPLC column was observed as a negative peak corresponding to its antioxidant activity.	HPLC-UV	Determination DPPH activity in apples	Bandoniene and Murkovic, 2002
	HPLC-UV	Determination DPPH activity in aromatic plants	Goulas et al., 2012, 2014
	HPLC-UV	Determination ABTS activity in aromatic plants	Jeon et al., 2011
**CHEMOMETRICS**			
Prediction of antioxidant activity from chromatographic fingerprints	HPLC/ partial least squares (PLS) models	Prediction of the TEAC capacity of green tea	Dumarey et al., 2008
Prediction of antioxidant activity in carrots on the basis of color data measured using Computer Vision System (CVS)	CVC/ multiple linear regression models	Prediction of the DPPH activity in carrots	Pace et al., 2013
Artificial neural networks (ANN) based model was designed and trained using the backpropagation algorithm for performing prediction of the DPPH activity. A series of chemical analysis was used for training.	Chemical analysis/ANN	Prediction of the DPPH activity in teas	Cimpoiu et al., 2011

a FTIR: Fourier Transform Infrared

b DPPH: 1,1-diphenyl-2-picryl-hydrazyl

c FRAP: ferric reducing/antioxidant power

d TEAC: Trolox equivalent antioxidant capacity

e NIR: Near-infrared

f ORAC: oxygen radical absorbance capacity

g ABTS: 2,2′-Azino-bis(3-ethylbenzothiazoline-6-sulfonic acid) diammonium salt

h EDTA: Ethylenediaminetetraacetic acid

i *CUPRAC: cupric ion reducing antioxidant capacity*.

### Infrared (IR) spectroscopy

Infrared (IR) spectroscopy is widely used for analysis, quality control and authentication in food research and industry. In general, it is a fast, accurate, easy and non-destructive technique that can potentially replace the classical chemical analyses. For quantitative purposes, a reliable calibration model needs to be developed (Zhang et al., 2004), followed by a partial least-squares regression calculations to predict the antioxidant activity of a given plant material; while the use of multiple linear regression, artificial neural network, and least squares support vector machine can be also applied (Lu and Rasco, 2012; Wu et al., 2012). Regarding band assignments in the IR region of spectra, hydroxyl and phenolic functional groups were most closely correlated with antioxidant capacity. Further, a close correlation exists between IR estimated values and *in vitro* assays, such as DPPH, FRAP, ABTS, and ORAC that has been applied to predict antioxidant activity in fruit (Lam et al., 2015), onion, shallot, garlic (Lu et al., 2011a,b) and medicinal plants (Bunaciu et al., 2012).

### Electron paramagnetic resonance (EPR) spectroscopy

Electron paramagnetic resonance (EPR) spectroscopy is the only analytical technique that can directly detect and quantify free radicals generated by *ex vivo* and *in vivo* chemical reactions. An EPR spectrum is the first derivative of the absorbance peaks when chemical species having unpaired electrons absorb radiation into the microwave range in a magnetic field, and the integration of the area under curve correlates with the number of radicals. Thus, the time-dependent scavenging of free radicals by the antioxidants can be monitored (Osorio et al., 2011). The antioxidant status of fresh produce can be effectively monitored through the elimination of stable free radicals (i.e., ABTS^∙+^, DPPH^∙^, or hydroxyl radicals (OH^∙^) added to the sample. Among the main advantages of EPR are a) the high sensitivity, allowing the detection at the submicromolar level, and b) the possibility to analyze turbid or highly colored samples (Sgherri et al., 2011). Opposite to colorimetric assays, EPR is more specific due to the formation of characteristic signals (Osorio et al., 2011).

Nowadays, EPR has been successfully applied to assess the radical scavenging activity of different foodstuffs rich in phenolic compounds. It can be also used to study the ability of hydrophilic as well as lipophilic antioxidants to scavenge free radicals. In particular, such applications of EPR have been described for fruit (blueberry, apple, corozo), aromatic plants (thyme, basil), tea and coffee samples (Morsy and Khaled, 2002; Pérez-Martínez et al., 2010; Osorio et al., 2011; Sgherri et al., 2011; Mirosavljević et al., 2015; Orłowska et al., 2015).

### Electrochemical methods

Electrochemical properties of pure antioxidants and/or plant foods can be exploited for the evaluation of their antioxidant activity. Electrochemical methods are based on the direct relation between expected antioxidant activity and electric oxidation potential. Among the electrochemical methods, the cyclic voltammetry (CV) technique has been adapted to determine the antioxidant activity (reducing capacity) of low molecular weight antioxidants (Magalhães et al., 2008). CV tracings are usually recorded from − 0.5 to + 1.5 V vs. the reference electrode at a scan rate of 100 or 400 mV s^−1^ (Chevion et al., 2000).

CV techniques allow rapid screening of the antioxidant activity of complex mixtures as foods, fruit and vegetables. In addition, the determination of antioxidant activity in aqueous medium as well as in organic solvents is feasible. Therefore, it is a useful and powerful tool to detect water and lipid soluble antioxidants (Chevion et al., 2000). Moreover, turbid or intensely colored samples can be measured without prior sample preparation, such as discoloration, centrifugation etc (Magalhães et al., 2008). In the last decade, numerous studies described the application of CV techniques to detect the antioxidant activity in horticultural commodities, such as herbs (Cosio et al., 2006), wild plants (Barros et al., 2011), berries (Cata et al., 2016), mushrooms (Barros et al., 2008), and onions (Sun-Waterhouse et al., 2008).

### Flow injection analysis (FIA) methods

Researchers also pay attention to FIA systems as these convert classic antioxidant assays to automated techniques. Their advantages are the ease of operation, the potential of miniaturization, the low consumption of the sample and reagents, the versatility and the high analytical frequency (Martins et al., 2013). FIA involves the rapid injection of a sample into a non-segmented, continuous carrier stream. Regarding instrumentation, the simplest set-up is a single line and merging zones that can be controlled manually, whereas the most advanced ones involve a multi-syringe configuration and/or computer-controlled sequential injection (Magalhães et al., 2007, 2009).

*In vitro* assays (i.e., ABTS, DPPH, and FRAP) and chemiluminescence have been adopted to FIA systems. The literature includes several interesting FIA systems to monitor the antioxidant activity in plant extracts, pure antioxidants and agricultural products like wine and honey (Shpigun et al., 2006; Blasco et al., 2007; Alvarez-Suarez et al., 2010; Pulgarín et al., 2012).

### Optical sensors

Being rapid, sensitive and low-cost screening applications, the optical sensors have recently become very popular (Steinberg and Milardović, 2007). They are based on the immobilization of chromogenic reagents (colorful radicals, reducing compounds and nanoparticles) onto suitable membranes, such as plasticized PVC films, Nafion membrane, paper and multilayer membranes (Çekiç et al., 2015; Choleva et al., 2015; Li et al., 2016). A number of sensors has been proposed to evaluate the intensity of coloration and to calculate thereafter the antioxidant activity in foods, including orange, apricot and peach juices, green and black tea, olive oil, coffee etc., (Steinberg and Milardović, 2007; Bener et al., 2010).

### High resolution screening (HRS) methods

High resolution screening (HRS) methods combine a chromatographic technique, such as HPLC or GC with a post-column (bio)chemical detection apparatus i.e., DAD, MS, and NMR. A major concern in HRS methods is to find an assay that is compatible in terms of time-scale and (bio)chemical conditions with HPLC separations (Niederländer et al., 2008). HRS methods produce a high resolution biochromatogram that can pinpoint the antioxidants of fresh produce. They are rapid, since fractionation and/or isolation procedures are omitted. Among these methods, HPLC-DPPH/ABTS assays have been successfully applied to study the antioxidant composition of apples (Bandoniene and Murkovic, 2002), aromatic plants (Goulas et al., 2012, 2014) and edible flowers (Jeon et al., 2011).

### Chemometrics

These methods are suitable to predict the antioxidant activity of complex matrices usually encountered in horticultural produce. Interestingly, the coupling of chromatographic fingerprints with chemometrics was applied to predict successfully the antioxidant activity in green tea and damiana herb (Dumarey et al., 2008; Lucio-Gutiérrez et al., 2012). Artificial neural networks were also employed to predict the antioxidant activity of herbs of known chemical composition (Cabrera and Prieto, 2010; Cimpoiu et al., 2011), while Pace et al. (2013) reported the combined use of multiple regression models with computer vision systems to predict antioxidant activity in carrots.

## Conclusions

The promotion of selected cultivars of high antioxidant potential for a given species and directing breeding strategies toward the development of high nutritional value cultivars is expected to increase the consumption of horticultural commodities. It should be also taken into consideration the fact that the massive use of commercial cultivars with improved yield attributes has possibly resulted in the loss of some quality traits, including antioxidant compounds, found in landraces and unexplored traditional cultivars with local interest. Moreover, marketing strategies should also give an extra value to growers by directing the health-concerned consumers to consume products of high antioxidant content.

The evaluation of antioxidant properties of fresh produce is usually performed by simple spectrophotometric assays. Furthermore, advanced analytical methods based on IR and EPR spectroscopy and electrochemistry have been developed, while HRS methods allow the determination of antioxidant activity of individual phytochemical in a complex matrix like horticultural produce. IR spectroscopy and optical sensors are recommended to estimate the antioxidant activity for industrial purposes as rapid and low cost procedures that can be automated.

## Author contributions

GAM organized and drafted this manuscript. IM, VG, and PD wrote selected parts of this review. All authors read and approved the manuscript.

### Conflict of interest statement

The authors declare that the research was conducted in the absence of any commercial or financial relationships that could be construed as a potential conflict of interest.
